# Glycerophosphate/Acylglycerophosphate Acyltransferases

**DOI:** 10.3390/biology3040801

**Published:** 2014-11-19

**Authors:** Atsushi Yamashita, Yasuhiro Hayashi, Naoki Matsumoto, Yoko Nemoto-Sasaki, Saori Oka, Takashi Tanikawa, Takayuki Sugiura

**Affiliations:** Faculty of Pharma-Sciences, Teikyo University, 2-11-1 Kaga, Itabashi-Ku, Tokyo 173-8605, Japan; E-Mails: hayashiy@pharm.teikyo-u.ac.jp (Y.H.); n-matsu@pharm.teikyo-u.ac.jp (N.M.); ynsasaki@pharm.teikyo-u.ac.jp (Y.N.-S.); s.oka@pharm.teikyo-u.ac.jp (S.O.); tanikawa@pharm.teikyo-u.ac.jp (T.T.); sugiurat@pharm.teikyo-u.ac.jp (T.S.)

**Keywords:** acyltransferase, acyl-CoA, triacylglycerol, phospholipid, GPAT, AGPAT

## Abstract

Acyl-CoA:glycerol-3-phosphate acyltransferase (GPAT) and acyl-CoA: 1-acyl-glycerol-3-phosphate acyltransferase (AGPAT) are involved in the *de novo* synthesis of triacylglycerol (TAG) and glycerophospholipids. Many enzymes belonging to the GPAT/AGPAT family have recently been identified and their physiological or pathophysiological roles have been proposed. The roles of GPAT/AGPAT in the synthesis of TAG and obesity-related diseases were revealed through the identification of causative genes of these diseases or analyses of genetically manipulated animals. Recent studies have suggested that some isoforms of GPAT/AGPAT family enzymes are involved in the fatty acid remodeling of phospholipids. The enzymology of GPAT/AGPAT and their physiological/pathological roles in the metabolism of glycerolipids have been described and discussed in this review.

## 1. Introduction—GPAT and AGPAT in Glycerolipid Metabolism

**Figure 1:**
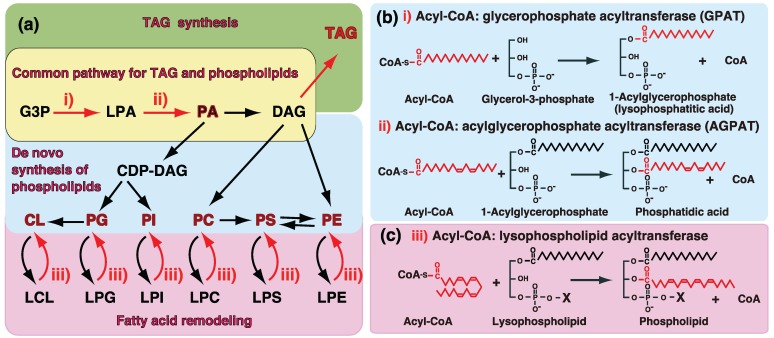
(**a**) Biosynthetic pathway of TAG and glycerophospholipids. GPAT (**i**) and AGPAT (**ii**) are involved in the common biosynthetic pathway of TAG and glycerophospholipids. After the* de novo* synthesis of phospholipids, individual phospholipids are subjected to fatty acid remodeling. Acyl-CoA:lysophospholipid acyltransferase (**iii**) reactions are involved in the fatty acid remodeling pathways of glycerophospholipids. The reactions of GPAT/AGPAT and acyl-CoA:lysophospholipid acyltransferase are shown in (**b**) and **(c)**.

TAG and glycerophospholipids are major constituents of glycerolipids [1,2,3,4,5,6,7]. TAG is involved in the storage and transport of fuels in lipid droplets and lipoproteins. In contrast, glycerophospholipids are the major constituents of biomembranes including plasma or organelle membranes. The *de novo* biosynthesis of glycerolipids is initiated by the incorporation of fatty acids into the glycerol backbone (Figure 1a). Phosphatidic acid (PA), also termed 1,2-diacylglyceropho-3-poshate (diacyl-GP), is known to be a common intermediate for the synthesis of TAG and glycerophospholipids [1,2,3,4,5,6,7]. GPATs and AGPATs are involved in the synthesis of PA (Figure 1a,b). PA is dephosphorylated to diacylglycerol (DAG) and DAG is further acylated in order to synthesize TAG (Figure 1a). Therefore, GPATs and AGPATs are important for the synthesis of TAG since most fatty acids are incorporated by these enzymes.

**Figure 2:**
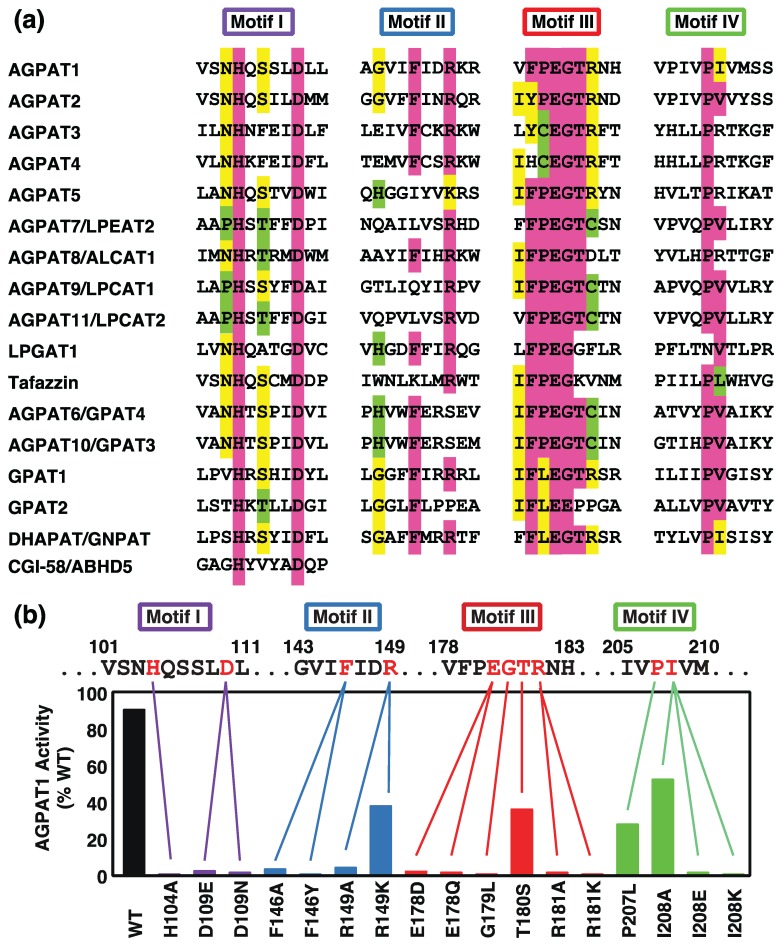
(**a**) Acyltransferase motifs in GPATs, AGPATs, tafazzin, and CGI-58. The conserved amino acid residues are shown in color; (**b**) Effects of amino acid substitutions at the human AGPAT1 acyltransferase motifs on AGPAT activity. The figure has been modified from Yamashita* et al.* [12].

Many enzymes belonging to the GPAT/AGPAT family have recently been identified [1,4,5,7]. These enzymes possess four well-conserved domains that have been suggested to play a role in the activities of acyltransferases (Figure 2a). Of these enzymes, four GPATs (GPAT1, GPAT2, GPAT3/AGPAT10, and GPAT4/AGPAT6) and at least two AGPATs (AGPAT1 and AGPAT2) are known to be involved in the *de novo* synthesis of TAG and phospholipids. Previous findings showed that the accumulation of TAG was closely related to obesity and obesity-related diseases, suggesting the physiological and pathological roles of GPAT/AGPAT. The identification of the causative genes of diseases and analyses of genetically manipulated animals clearly implicated GPAT/AGPAT in these diseases [1,5,7].

Individual phospholipids are synthesized through the common precursor, PA; phosphatidylcholine (PC) and phosphatidylethanolamine (PE) are synthesized via CDP-choline/CDP-ethanolamine pathways while phosphatidylinositol (PI) and cardiolipin (CL) are synthesized via the CDP-DAG pathway (Figure 1a). Since all fatty acids are incorporated by GPAT and AGPAT in the *de novo* synthesis of glycerolipids, the acyl-CoA specificity of GPAT/AGPAT determines the fatty acid composition of not only PA, but also *de novo* synthesized glycerophospholipids [1,2,3,4,5,6,7,8].

The fatty acid compositions of mature phospholipids are known to be markedly different from those of *de novo* synthesized glycerophospholipids. The fatty acid compositions of individual phospholipids are regulated after their *de novo* synthesis, *i.e.*, by a fatty acid remodeling system. Polyunsaturated fatty acids such as arachidonic acid are not commonly introduced into phospholipids through *de novo* synthesis, but have been shown to be incorporated during the fatty acid remodeling of phospholipids [2,3,4,5,6,7,8]. Previous studies reported that acyl-CoA:lysophospholipid acyltransferase reactions were involved in this remodeling system. Enzymes belonging to the membrane-bound *O*-acyltransferase (MBOAT) family involve the fatty acid remodeling of phospholipids [5,7,8]. Furthermore, some isoforms of GPAT/AGPAT family enzymes possess acyltransferase activity towards lysophospholipids such as lysophosphatidylcholine (LPC), suggesting that AGPAT enzymes also contribute to the fatty acid remodeling of glycerophospholipids (AGPAT7-9 and AGPAT11).

Because of recent advances in research involving GPAT/AGPAT family enzymes, several excellent review articles have been published [4,5,6,7,8]. In this review, we first focus on the catalytic roles and substrate recognition of acyltransferase motifs conserved in GPAT/AGPAT family enzymes (Section 2). Recent evidence suggests the involvement of GPAT/AGPAT enzymes in obesity and related diseases, and we also focus on the physiological and pathological roles in TAG synthesis (Section 3 and Section 4). Finally we discuss another important role of AGPATs, *i.e.*, the fatty acid remodeling of phospholipids (Section 5). In this review, fatty acids are sometimes designated in terms of the number of carbon atoms: the number of double bonds; e.g., 20:4 denotes arachidonic acid.

## 2. Acyltransferase Motifs Conserved in GPAT/AGPAT Family Enzymes

The sequence alignment of GPAT/AGPAT family enzymes revealed the existence of several regions of strong homology, acyltransferase motifs I-IV, as shown in Figure 2a [4,5,7]. Since all enzymes catalyze the transfer of a fatty acid from acyl-CoA to the glycerol backbone, these motifs provide the potential to recognize common or similar substrate structural elements and furnish the catalytic machinery for acyltransferase reactions. Catalytically important amino acids were determined using site-directed mutagenesis in the acyltransferase motifs of *E. coli* GPAT and murine GPAT1 as models [9,10,11]. We examined the catalytic roles of highly conserved residues in the acyltransferase motifs of human AGPAT1 (Figure 2b) [12]. The roles of the motifs in LPCAT1/AGPAT9 were also examined [13].

### 2.1. Acyltransferase Motif I

Acyltransferase motif I is the most conserved region among GPAT/AGPAT family enzymes [8,9,10,11]. All enzymes possess the HXXXXD signature (Figure 2a). In human AGPAT1, the mutation of Asp109 to asparagine or glutamate resulted in the complete loss of AGPAT activity (Figure 2b). Another substitution in motif I, H104A, also abolished this activity. In *E. coli* GPAT, the mutation of H306G or D311G markedly reduced the *Vmax*, but did not significantly alter *Km* value [10]. In mouse LPCAT1/AGPAT9, the substitution of H135 or D to A resulted in the complete loss of the activity [13].

In a model for *E. coli* GPAT catalysis [9,10], the invariant histidine in the HXXXXD signature acted as a general base to abstract a proton from the hydroxyl group at the sn-1 position of glycerol-3-phospate (G3P), thereby facilitating a nucleophilic attack on the acyl-CoA thioester. The invariant aspartate in the HXXXXD signature may act in a charge relay system with the invariant histidine residue to increase the nucleophilicity of the G3P hydroxyl group. The model ascribed a catalytic role to motif I. This model is supported by the finding that plant GPATs lacking motifs III and IV could catalyze acyltransferase activity [10,14]. In addition, CGI-58 (the causative gene product for Chanarin-Dorfman syndrome) and its related proteins possessed the HXXXXD signature only, but could catalyze acyltransferase activity, suggesting that the HXXXXD signature is important for the activity [15].

### 2.2. Acyltransferase Motif II

Phenylalanine to alanine (F146A) or tyrosine (F146Y) substitutions in human AGPAT1 motif II had marked effects on AGPAT activity (Figure 2b). The R149A mutant had no AGPAT activity, whereas the conservative mutant, R149 K, retained a large amount of AGPAT activity. Therefore, a positive charge appears to be important at position 149.

We proposed that motif II may be involved in the acyl receptor binding. In human AGPAT1, the amino acid substitution at motif II (R149 K) reduced affinity for LPA, but not that for acyl-CoA [12]. In* E. coli* GPAT, mutations in motif II reduced affinity for G3P [10], suggesting that motif II is involved in the binding of G3P. These results strongly indicated that the conserved motif II may be involved in the binding of acyl acceptor LPA and G3P. However, in mouse LPCAT1, Harayama* et al.* proposed the motif II may be binding site of acyl-CoA, based on the different activity with acyl- or acetyl-CoA by the site-directed mutagenesis in the region [13].

### 2.3. Acyltransferase Motif III

Motif III is the most conserved region in GPAT/AGPAT family enzymes. Most of these enzymes possess the PEGT-X signature (Figure 2a). In human AGPAT1, E178Q and E178D mutations in the signature removed AGPAT activity, suggesting that both the negative charge and sidechain length at this position were important for AGPAT activity (Figure 2b) [12]. In the PEGT-X signature of mouse GAPT1, the substitution of E315 to A or Q markedly reduced but not complete lost the activity [11], suggesting that the requirement of glutamate in this position is strict in human AGPAT1 compared to mouse GPAT1.

In the PEGT-X signature of human AGPAT1, the G179L mutant also lacked AGPAT activity, suggesting that the small amino acid sidechain here was essential for this activity [12]. In mouse LPCAT1, the substitution of G209 to A slightly reduced the activity, suggesting that methyl residue may be exchangeable in this sidechain [13].

In human AGPAT1, threonine 180 could be changed to a serine with the retention of a large amount of AGPAT activity, suggesting that a hydroxy residue is important at this position [12].

In the PEGT-X signature, the subfamily enzymes including AGPAT1 possess arginine at the X position. The subfamily includes AGPAT1-5, GPAT1, and DHAPAT. In human AGPAT1, alanine or lysine substitutions for Arg181 in motif III resulted in the loss of AGPAT activity (Figure 2b) [12]. The similar requirement of arginine in this position was observed in mouse GPAT1 [11]. The lack of this activity in the R181K mutant (human APGAT1) or R318K mutant (mouse GPAT1) suggested that arginine itself, and not just a positive charge, was essential at this position. However, some enzymes including LPCAT1 and LPCAT2 possess cysteine at the X position of the PEGT-X signature, and the roles of cysteine have not yet been fully established in this motif.

Regarding the function of Motif III, some *E. coli* GPAT mutant in motif III was shown to reduce affinity for G3P [10]. We also reported that a T180S mutation in human AGPAT1 reduced affinity for LPA, but not for acyl-CoA [12]. Taken together, these findings indicate that motif III is also involved in the binding of the acyl acceptor, *i.e.*, G3P for GPAT and LPA for AGPAT, respectively.

### 2.4. Acyltransferase Motif IV

Motif IV is less conserved among the four acyltransferase motifs and is rich in hydrophobic amino acids. In human AGPAT1, the mutation of Pro207 to leucine had modest effects on AGPAT activity (Figure 2b). The substitution of Ile208 with alanine had modest effects, whereas the substitution with glutamate or lysine, both charged residues, completely abolished AGPAT activity. The *Km* for palmitoyl-CoA was increased in the P207L and I208A mutants, suggesting that motif IV may participate in acyl-CoA binding [12].

### 2.5. Substrate Specificity and Membrane Topology of Acyltransferase Motifs

We previously proposed that the membrane topology of acyltransferase motifs was an important determinant for the substrate accessibility and specificity of GPAT/AGPAT [11]. AGPAT1 and AGPAT2 are located in the ER membrane and their substrate (acyl acceptor) is LPA. GPAT1 and GPAT2 are located in the outer membrane of mitochondria, whereas GPAT3 and GPAT4 are in the ER membrane, and their substrate (acyl acceptor) is G3P. Both acyl acceptors are chemically similar because of the presence of the common structure of G3P, with the only difference being the presence or absence of a fatty acyl (hydrophobic) residue.

**Figure 3:**
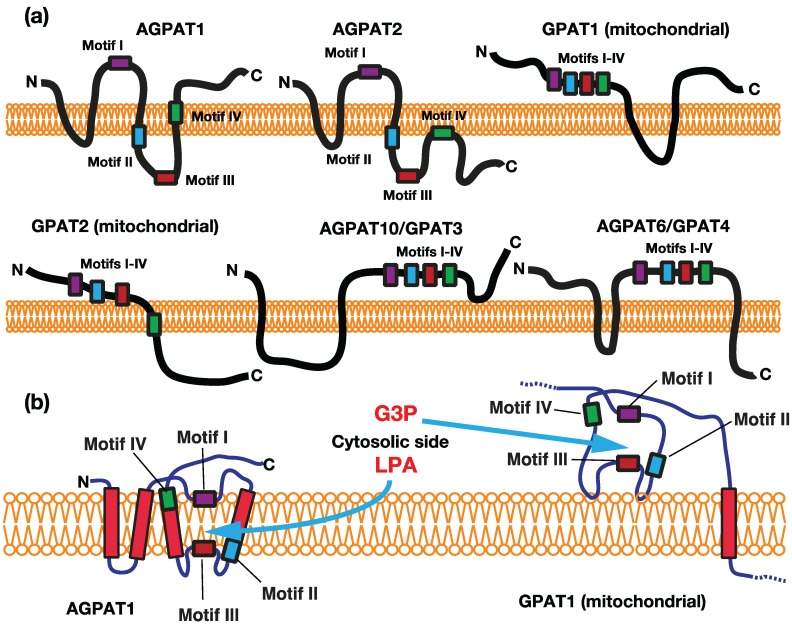
(**a**) Predicted transmembrane segments and relationship to the acyltransferase motifs of GPAT/AGPAT family enzymes. The transmembrane segments of each enzyme were predicted by several algorithms including TMHMM and SOSUI. The locations of acyltransferase motifs I-IV have also been depicted; (**b**) Proposed models of the catalytic sites of AGPAT1 and GPAT1. The top or bottom sides of the membrane show the cytosol or lumen (interspace) of the ER and mitochondrial outer membranes.

In the case of GPATs, the acyltransferase motifs, except for motif IV of GPAT2, are predicted to be exposed on the cytosolic side of the mitochondrial outer membrane or ER (Figure 3a). Motifs II/III, which were suggested to be involved in the binding of the acyl acceptor [10], are all located in the cytosol. The catalytic site of GPATs should be in the cytosol, as depicted in Figure 3b, which is consistent with the finding that hydrophilic G3P could access the catalytic domain of the enzyme.

In contrast, human AGPAT1 and AGPAT2 were predicted to have 3 or 4 transmembrane domains, with the acyltransferase motifs being separated by the transmembrane segment, placing motif I on the cytosolic side and motifs II and III on the luminal side (Figure 3). This topology of motifs I and III of AGPAT1 was confirmed by the findings of glycosylation experiments [12]. Since all motifs as well as transmembrane segments are expected to comprise the catalytic machinery, we proposed that the catalytic site is located within the membranes due to the penetration of loops carrying motifs I or motifs II/III into the membranes from both sides, as depicted in Figure 3b. We considered motifs I and IV to be near the cytosolic surface and motifs II and III to be deep in the membrane.

We previously examined the substrate accessibility of human AGPAT1 [12]; LPA, but not G3P could access the catalytic site. The hydrophobicity of LPAs with fatty acids longer than twelve carbons was necessary for accessing the catalytic site. The LPA binding site could not be reached by more polar LPAs or by molecules that lacked hydrophobic moieties, such as G3P. This was consistent with the proposal that the hydrophobicity of LPA may be necessary to reach the LPA binding site deep in the hydrophobic environment of the membrane.

## 3. Glycerophosphate Acyltransferases (GPATs)

GPATs (EC 2.3.1.15) catalyze the first step in the synthesis of glycerolipids and convert G3P to LPA (1-acyl-GP) by the transfer of a fatty acid from acyl-CoA (Figure 1a,b (i)). Because GPAT exhibits the lowest specific activity of enzymes, it is rate limiting in the *de novo* pathway of TAG and glycerophospholipids [1,2,3,4,5,6,7,8]. Two biochemically different GPATs were previously identified; mitochondrial and microsomal GPATs. Mitochondrial GPAT is resistant to inactivation by sulfhydryl agents such as NEM and prefers palmitoyl-CoA to oleoyl-CoA as an acyl donor, while microsomal GPAT is NEM-sensitive and has no preference for palmitoyl-CoA. The properties and tissue distributions of GPATs are summarized in Table 1 and Table 2.

### 3.1. Mitochondrial GPATs (GPAT1 and GPAT2) 

Two mitochondrial GPATs have so far been identified. Mouse Gpat1 was first cloned as a gene product that was highly expressed in mouse lipogenic tissues, such as the liver and adipose tissue, and was markedly induced by fasting/refeeding and the administration of insulin [16,17,18]. Based on similarities in its sequence to that of *E. coli* GPAT, the gene was identified as mammalian GPAT1.

GPAT1 localized to the mitochondrial outer membrane and its activity was resistant to NEM, which was consistent with previously characterized mitochondrial GPAT activity. 

Biochemical studies revealed that saturated fatty acyl-CoAs were preferred approximately two-fold more than unsaturated fatty acyl-CoAs [19]. Gpat1 catalyzed selective fatty acid transfer to the sn-1 position of G3P. The preference for C16:0-CoA by Gpat1 was confirmed in mice with the knockdown of hepatic Gpat1; reduced amounts of C16:0 and increased amounts of C18:0 and C18:1 were observed in liver PC and PE [20,21]. In contrast, the overexpression of Gpat1 in the livers of mice or rats led to the increased incorporation of C16:0 fatty acids into LPA, DAG, and TAG [22,23]. PC and PE in the livers of Gpat1^−/−^ mice contained 40% more C20:4 at the *sn*-2 position, suggesting that fatty acid species at the *sn*-2 position were influenced by fatty acids at the *sn*-1 position [20]. The analysis of hepatocytes from Gpat1^−/−^ mice also revealed that GPAT1 was required to incorporate *de novo* synthesized fatty acids into TAG and divert them from oxidation [24].

GPAT1 activity is known to be transcriptionally regulated. Previous studies showed that GPAT1 expression levels were the highest in adipose tissue and the liver, followed by muscle, the brain, kidneys, and lungs [1,25]. In most tissues, only 10% of all GPAT activity can be attributed to mitochondrial GPAT. However, mitochondrial GPAT constitutes 30%–50% of all activity in the liver and has a large impact on the regulation of TAG synthesis [1]. The induction of Gpat1 mRNA by insulin was shown to be mediated by sterol regulatory element-binding protein-1, and glucagon counteracted the actions of insulin by elevating cAMP levels [26]. The agonist for a nuclear receptor (liver X receptor, LXR) also increased Gpat1 mRNA levels [27].

Gpat1 enzyme activity was also found to be post-transcriptionally regulated by insulin and AMP-activated protein kinase (AMPK). The treatment of rat primary adipocytes with insulin acutely increased the Km and Vmax of Gpat1 for its substrates, and this was mediated through protein phosphorylation [28]. In contrast, AMPK, a sensor of the availability of cellular energy, inhibited GPAT1 activity [1,26].

A novel function has been proposed for GPAT1. GPAT1 and the ortholog of *C. elegans* were shown to be essential for mitochondrial fusion [29]. The mutation of* C. elegans* mitochondrial GPAT caused excessive mitochondrial fragmentation, and this defect was rescued by the accumulation of LPA, the product of GPAT. The depletion of human GPAT1 also impaired the mitochondrial fusion process in HeLa cells. These findings demonstrated that LPA produced by mitochondrial GPAT functioned as an essential factor in mitochondrial fusion.

GPAT2 was first identified as another mitochondrial enzyme due to the remaining mitochondrial GPAT activity in Gpat1^−/−^ mice [30]. In contrast to Gpat1, Gpat2 activity was sensitive to NEM and heating at 40.5 °C, was inhibited by dihydroxyacetone phosphate and polymyxin B, and lacked a preference for C16:0-CoA. Furthermore, GPAT2 activity constituted 60% of all GPAT activity in purified mitochondria from the wild-type mouse liver. cDNA cloning of GPAT2 was based on amino acid sequence similarities to GPAT1 [25,31]. The GPAT2 active site region has similar motif I and IV sequences and markedly different motif III sequences to GPAT1 (Figure 2).

**Table 1:** Summary of GPAT/AGPAT family enzymes.

Symbol	Other Symbol	Acyl Acceptor	Acyl donor (Acyl-CoA)	Notes	Diseases in Human, Phenotype of Gene-Manipulated Animal
GPAT1		G3P	16:0 > 18:1n-9 (Preference to saturated species)	Mitochondrial GPAT, NEM-resistant	
GPAT2		G3P	No preference to saturated species	Mitochondrial GPAT, NEM-sensitive	
GPAT3	AGPAT8, AGPAT10	G3P	No preference to saturated species	Microsomal GPAT, NEM-sensitive	
GPAT4	AGPAT6	G3P	No preference to saturated species	Microsomal GPAT, NEM-sensitive	Causative gene of lipodystrophy
DHAPAT	GNPAT	DHAP		Biosynthesis of ether-linked phospholipids	Causative gene of TYPE 2 rhizomelic chondrodysplasia punctata
AGPAT1	LPAATα, LPAAT1	LPA			
AGPAT2	LPAATβ, LPAAT2	LPA			Causative gene of lipodystrophy
AGPAT3	LPAATγ, LPAAT3	LPA. LPI	Preference to PUFA species		
AGPAT4	LPAATδ, LPAAT4	LPA		Location of mitochondria?	
AGPAT5	LPAATε, LPAAT5			Location of mitochondria?	
AGPAT7	LPAATη, AYTL3, LPEAT2	LPE	Preference to oleic acid		
AGPAT8	ALCAT1, LCLAT1, LYCAT1	Lysocardiolipin, LPG, LPI (1-acyl or 2-acyl)	PUFA for LCL, 18:0 for 2-acyl LPI		Mitochondrial dysfunction associated with hypertrophic cardiomyopathy, blood lineages
AGPAT9	LPCAT1, AYTL2	LPC, LPE, LysoPAF	Preference to saturated species, Acetyl-CoA	Surfactant biosynthesis, EF-hand motif	
AGPAT11	LPCAT2, AYTL1, LysoPAFAT	LPC, LPE, LysoPAF	Acetyl-CoA, Acyl-CoA	Inducible enzyme for PAF biosynthesis, EF-hand motif	
LPGAT1		LPG			
TFZ	Tafazzin	Lysocardiolipin			Causative gene of Barth syndrome

The tissue distribution of Gpat2 was also distinct from Gpat1. The expression of mouse Gpat2 was 50-fold higher in the testis than in the liver, with even lower expression in other tissues including adipose tissue, skeletal muscle, the brain, adrenal glands, kidneys, lungs, and the heart [25]. Gpat2 mRNA did not increase in the livers of rats re-fed a high-sucrose diet after fasting, suggesting that Gpat2 did not contribute to the diet-induced synthesis of hepatic TAG [25].

**Table 2:** Tissue distribution of GPAT/AGPAT family enzymes.

Symbol	Tissue Distribution
GPAT1	BAT > WAT > liver > muscle > brain
GPAT2	testis > liver > adipose tissue, skeletal muscle, brain, adrenal grand, kidney, lung, heart
GPAT3/AGPAT10	adipose tissue > small intestine > heart > brain > liver
GPAT4/AGPAT6	brown adipose tissue, testis > liver, kidney, brain, intestine, WAT > heart, skeletal muscle
AGPAT1/LPAATα	testis > spleen, thymus, prostate, ovary, small intestine, colon, PBL (nearly ubiquitous)
AGPAT2/LPAATβ	liver > pancreas >lung, heart, small intestine, skeletal muscle > colon, PBL > spleen, prostate >> brain
AGPAT3/LPAATγ	testis > kidney > liver > heart > brain
AGPAT4/LPAATδ	brain > skeletal muscle > spleen
AGPAT5/LPAATε	testis > prostate > placenta > brain
AGPAT7/LPEAT2	brain > stomach > heart > liver
AGPAT8/ALCAT1	heart, liver, kidney > small intestine, skin, brain, lung > spleen, thymus, testis > muscle, stomach
AGPAT9/LPCAT1	lung (alveolar type II cells) >> spleen > brain, heart, skeletal muscle, ovary, pancreas
AGPAT11/LPCAT2	macrophage >> neutrophil >> skin > brain, heart, stomach, colon, spleen > lung, liver, ovary, placenta
LPGAT1	liver, placenta > peripheral blood, lung, kidney, brain >> colon
	BAT; brown adipose tissue, WAT; white adipose tissue, PBL; peripheral blood leukocytes

### 3.2. Microsomal GPATs—GPAT3/AGPAT10 and GPAT4/AGPAT6

GPAT3 and GPAT4 are ER membrane isoforms that were initially designated as AGPAT8 [32,33] and AGPAT6 [34,35,36,37,38], respectively. The catalytic activities of GPAT3 and GPAT4 were found to be sensitive to NEM, which is consistent with previously characterized microsomal GPAT activity. Both proteins have all four acyltransferase motifs within an approximately 100 amino acid domain (Figure 2).

GPAT3 was first identified as a member of the AGPAT family (LPAAT-theta/AGPAT8) [32,34,35]. AGPAT8 was shown to localize to the ER. However, this enzyme did not exhibit AGPAT activity; the protein was instead found to have GPAT activity with NEM sensitivity [33]. These findings clearly showed that AGPAT8 was a microsomal type of GPAT and was consequently renamed GPAT3. Sukumaran *et al.* reported that the same enzyme possessed AGPAT activity and was renamed AGPAT10/GPAT3 [39]. They proposed that AGPAT10/GPAT3 catalyzed the sequential reactions of GPAT and AGPAT. GPAT3 employed a broad range of long-chain acyl-CoAs, including saturated and unsaturated species. The acyltransferase activity of GPAT3 was not detected with other lipid acceptors including MAG, DAG, or lysophospholipid substrates. 

GPAT3 was identified as the predominant GPAT in adipocytes because Gpat3 mRNA increased ~60-fold during the differentiation of 3T3-L1 cells to mature adipocytes [33] and total GPAT activity in the white adipose tissue of Gpat3^−/−^ mice was reduced by 80% [40]. The induction of GPAT4 was only modest (approximately 5-fold) [41]. Gpat3 mRNA in white adipose tissue was increased by the treatment of *ob/ob* mice with rosiglitazone, a PPARγ agonist, suggesting that Gpat3 is a PPARγ target gene in white adipose tissue and may contribute to increased insulin sensitivity in *ob/ob* mice treated with rosiglitazone [33]. The overexpression of GPAT3 in HEK293 cells increased the phosphorylation of p70 S6 kinase and 4E-binding protein 1 in an mTOR-dependent manner, suggesting the involvement of lipid intermediates of GPAT3, such as LPA and PA, in the mTOR pathway [32]. GPAT3 and GPAT4 were shown to be phosphorylated by insulin at the Ser and Thr residues, leading to increased GPAT activity that was sensitive to wortmannin [41].

In the liver, the deletion of GPAT3 had no impact on total GPAT activity, but resulted in a 30% reduction in NEM-sensitive GPAT activity. When female Gpat3^−/−^ mice were fed a high-fat diet, body weight gain and adiposity decreased while energy expenditure increased; however, the mutant mice were viable and fertile and exhibited no obvious metabolic abnormalities when fed standard laboratory chow [40]. A GPAT3 deficiency lowered fed, but not fasted, glucose levels and slightly improved glucose tolerance in diet-induced obese male and female mice. Gpat3^−/−^ mice fed a high-fat diet had enlarged livers and dysregulated cholesterol metabolism.

GPAT4 was also initially identified as a member of the AGPAT family (LPAAT-zeta /AGPAT6) based on the high similarity of its amino acid sequence to that of AGPATs [34,35,36]. The enzyme AGPAT6 was shown to be located in the ER. However, it did not exhibit AGPAT activity. After a careful examination of its enzyme activity, AGPAT6 was found instead to have GPAT activity. AGPAT6 was postulated to be a second ER-localized GPAT and was renamed GPAT4 [37,38]. The overexpression of GPAT4 in cultured cells resulted in increased NEM-sensitive GPAT activity without any preference for saturated fatty acyl-CoA, but not in increased AGPAT, DGAT, or other lysophospholipid acyltransferase activity.

In GPAT4^−/−^ mice, NEM-sensitive GPAT activity was reduced by 65% in the liver and brown adipose tissue, but was normal in gonadal white adipose tissue, suggesting a critical role for this isoform in the liver and brown adipose tissue [36]. The expression of Gpat4 was reported to be induced in the mammary gland epithelium during lactation, and the Gpat4^−/−^ mouse exhibited severely impaired lactation with reduced numbers of fat droplets in the mammary gland, suggesting a critical role for GPAT4 in the synthesis of TAG in mother’s milk [35]. The body weight of Gpat4^−/−^ mice fed a chow diet was 25% less than that of wild-type animals, and these mice were also found to be resistant to diet-induced obesity and genetic obesity on the *ob/ob* background [36].

The overexpression of GPAT4 in mouse hepatocytes impaired glucose homeostasis such as insulin-suppressed gluconeogenesis and insulin-stimulated glycogen synthesis [42]. Impaired glucose homeostasis was coupled to the inhibited insulin-stimulated phosphorylation of Akt-Ser473 and Akt-Thr308. GPAT4 overexpression inhibited the association between rictor and mTOR and mTORC2 activities, and also increased PA, especially di16:0-PA. In contrast, both the mTOR/rictor association and mTORC2 activity increased in Gpat4^−/−^ hepatocytes, while the content of PA was lower than in controls, with the greatest decrease being detected in 16:0-containing PA species. Liver and skeletal muscle from Gpat4^−/−^ mice fed a high-fat diet were more insulin sensitive and had a lower hepatic content of di16:0-PA. The GPAT4-derived lipid signal may impair insulin signaling in the mouse liver and contribute to hepatic insulin resistance.

### 3.3. Dihydroxyacetone Phosphate Acyltransferase (DHAPAT/GNPAT)

Dihydroxyacetonephosphate (DHAP) acyltransferase (DHAPAT, EC 2.3.1.42), also known as glyceronephosphate acyltransferase (GNPAT), is a member of the glycerophospholipid acyltransferase family [43,44,45,46]. This enzyme catalyzes the acylation of DHAP to form 1-acyl-DHAP, is localized exclusively within peroxisomes, and is involved in the biosynthesis of ether-linked phospholipids. DHAPAT cDNA was isolated based on the peptide sequences from purified enzymes [43,44] and encodes a 680-amino acid peptide containing a C-terminal type 1 peroxisomal targeting signal (PTS1). A genetic disorder, TYPE 2 rhizomelic chondrodysplasia punctata, has been linked to mutations in motif II and the deletion of DHAPAT [45,46]. These patients have impaired plasmalogen biosynthesis.

## 4. Acylglycerophosphate Acyltransferases (AGPATs) Involved in the *de novo* Synthesis of TAG and Phospholipids

The second acylation step in the glycerol phosphate pathway involves the conversion of LPA to PA, transferring an acyl group from fatty acyl-CoA to the *sn*-2 position of the glycerol backbone (Figure 1). AGPAT enzymes (EC 2.3.1.51) are involved in this reaction. AGPAT1 and AGPAT2 are well-established AGPATs, the enzyme activities of which have been validated [47,48,49,50,51]. The properties and tissue distributions of AGPATs are also summarized in Table 1 and Table 2.

### 4.1. AGPAT1/LPAATα

AGPAT1 was cloned and identified on the basis of sequence homology to* E. coli* and yeast AGPATs [47,48,50,51]. Mouse Agpat1 mRNA was found to be ubiquitously expressed in most tissues, including skeletal muscle, the heart, liver, lungs, kidneys, white and brown adipose tissue, brain, spleen, and thymus. Human and mouse AGPAT1 have a calculated molecular mass of 32 kDa. AGPAT1 was mainly located in the ER, but not in other subcellular compartments. The substrate specificity of human AGPAT1 was examined using a recombinant enzyme expressed in *E. coli*, mammalian cells, and insect cells. AGPAT1 exhibited acyl acceptor specificity for LPA with the absence of activity for other lysophospholipids, but showed broad acyl donor specificity for acyl-CoA with a preference for fatty acid species C12-16:0, C16:1, C18:2, and C18:3, followed by C18:0, C18:1, and C20:4, and poor activity for C20:0 and C24:0 [47,48,49,50,51,52].

Previous studies on the roles of AGPAT1 in adipose tissue and muscle were of importance because these tissues are derived from common precursor cells [53,54]. The overexpression of AGPAT1 in 3T3-L1 adipocytes and C2C12 myotubes increased exogenous oleate uptake and incorporation into TAG through PA [53]. AGPAT1 overexpression in 3T3-L1 adipocytes also increased insulin-stimulated glucose uptake and its conversion to lipids. In C2C12 myotubes, the overexpression of AGPAT1 did not alter glucose uptake in C2C12 myotubes, but influenced the metabolic fate of glucose, decreasing glycogen synthesis by 30% and increasing lipid synthesis by 33%. These findings indicated the similar, but different roles of AGPAT1 in energy handling between adipose tissue and muscle. In contrast, AGPAT1, but not AGPAT2, was suggested to be involved in skeletal muscle development [54].

### 4.2. AGPAT2/LPAATβ

Human AGPAT2 shows 35% amino acid identity to AGPAT1 [4,7,49]. The molecular masses of human and mouse AGPAT2 were both calculated to be 31 kDa. AGPAT2 was also located in the ER, but not in other subcellular organelles. The substrate specificities and preferences of AGPAT2 have been examined in detail [55]; AGPAT2 exhibited strict acyl acceptor specificity for LPA. However, AGPAT2 exhibited activity for lysophosphatidylmethanol, a membrane-permeable LPA analog, whereas AGPAT1 did not. AGPAT2 also showed broad acyl donor specificity for acyl-CoA with a preference for fatty acid species including C14:0, C16:0, C18:1, and C18:2 fatty acids, with the lower incorporation of C18:0 and C20:4-CoAs [49,55].

High levels of mouse Agpat2 mRNA have been detected in visceral white and brown adipose tissue, the liver, heart, and pancreas, with moderate levels in muscle, the lungs, small intestine, kidneys, and spleen, and low levels in the brain, placenta, and subcutaneous fat [36,48,55]. The critical roles of AGPAT2 in the synthesis of TAG in human adipocytes were established from studies on patients with mutations in the AGPAT2 gene, which causes congenital generalized lipodystrophy (CGL) [56].

CGL is an autosomal recessive disorder characterized by a marked lack of adipose tissue since birth, severe insulin resistance, hypertriglyceridemia, hepatic steatosis, and the early onset of diabetes. Most AGPAT2 mutations underlying CGL have been shown to cause the near-complete loss of AGPAT activity* in vitro* [57]. Consistent with the near-complete absence of adipose tissue, CGL patients have extremely low serum leptin and adiponectin levels [56,57,58,59]. Hypoleptinemia contributes to metabolic complications such as insulin resistance, hypertriglyceridemia, and hepatic steatosis.

AGPAT2 may also have a role in the differentiation of adipocytes and defects in this process may also result in lipodystrophy. The siRNA-mediated knockdown of AGPAT2 in OP9 adipocytes decreased the gene expression of adipogenic proteins such as PPARγ and CAAT/enhancer-binding protein (C/EBPβ) and delayed the induction of mature adipocyte markers, such as aP2 and GLUT4, in addition to reducing the accumulation of TAG [60]. The knockdown of AGPAT2 also suppressed the induction of other AGPAT genes and increased the levels of several phospholipid species during the differentiation of adipocytes. These findings suggested that AGPAT2 modulated cellular phospholipid levels, which may, in turn, influence the adipocyte differentiation program. The overexpression of AGPAT1 and AGPAT2 was shown to markedly enhance the IL-1β-stimulated release of IL-6 and TNF-α and significantly increased IL-6 and TNF-α mRNA levels in ECV304 cells, suggesting that AGPAT overexpression amplified cellular signaling from cytokines [47]. A similar mechanism may be involved in differentiation and adipokine synthesis in adipocytes.

Cortes* et al.* generated AGPAT2 null mice to elucidate the molecular mechanisms underlying the metabolic complications associated with an AGPAT2 deficiency [61]. AGPAT2^−/−^ mice developed severe lipodystrophy, which affected both white and brown adipose tissue, extreme insulin resistance, diabetes, and hepatic steatosis. The expression of lipogenic genes and rates of *de novo* fatty acid biosynthesis were 4-fold higher in AGAPT2^−/−^ mouse livers. The mRNA and protein levels of monoacylglycerol acyltransferase (MGAT) isoform 1 were markedly increased in the livers of AGPAT2^−/−^ mice, suggesting that an alternative MAG pathway for TAG biosynthesis was activated in the absence of AGPAT2. Liver triglyceride levels in AGPAT2^−/−^ mice fed a fat-free diet were reduced by 50%. These findings suggested that both dietary fat and hepatic TAG biosynthesis via the MAG pathway may contribute to hepatic steatosis in AGPAT2^−/−^ mice.

Several lines of evidence have been reported concerning the relationship between the increased expression of AGPAT2 and several cancers,* i.e.*, the augmented expression of AGPAT2 were observed in some human cancers [62,63,64,65,66,67,68,69]. AGPAT2 inhibitors have been shown to induce growth arrest, apoptosis, or necrosis in various tumor cell lines [62,63,64,65,66,67]. Inhibition of AGPAT2 block Ras/Raf/Erk and PI 3-kinase/Akt pathways, suggesting the involvement of PA produced by AGPAT2 in these pathways [62]. The antitumor activities of AGPAT2 inhibitors were also demonstrated in mice bearing tumor xenografts [64,65]. Elevated AGPAT2 expression has been associated with the aggressive histology and poor prognosis of ovarian cancer [65,68,69]. These observations promise that AGPAT2 is a potential target of cancer therapy.

### 4.3. AGPAT3/LPAATγ

AGPAT3 was first identified as one of the AGPAT family enzymes [70,71,72]. AGPAT3 mRNA in the mouse heart appeared to be regulated by the activation of PPAR-α [70]. Yuki* et al.* found that mouse AGPAT3 was highly expressed in the testis [73] in a manner that was dependent on the age of the mouse, with increased expression levels being observed in the β-estradiol-treated testicular cell line, TM4 Sertoli cells, suggesting that AGPAT3 was regulated by sex hormones and may be involved in the maturation of the testis [73]. Mouse AGPAT3 predominantly localized to the ER. Human AGPAT3 was also located in the nuclear envelope; however, the physiological meaning of the nuclear localization of this enzyme has not yet been established [72]. Schmidt and Brown also reported that AGPAT3 was present in the Golgi apparatus [74].

Mouse AGPAT3 exhibited LPAAT activity with a preference for arachidonoyl(20:4n-6)-CoA and docosahexanoyl(22:6n-3)-CoA as a donor [73,75,76]. AGPAT3 was shown to be involved in the formation of polyunsaturated fatty acid (PUFA)-containing PC in TM4 Sertoli cells. PUFA-containing PC may have been formed through PUFA-containing PA by AGPAT3 (PUFAs may be incorporated via the *de novo* pathway). The induction of AGPAT3 during germ cell development critically contributed to the accumulation of PUFAs in testicular phospholipids, and may have affected sperm cell production. However, AGPAT3 also exhibited acyltransferase activity toward other lysophospholipids such as LPC, LPI, and LPS with a significant preference for PUFA-CoA. This enzyme may also be involved in both the *de novo* synthesis and remodeling of phospholipids; however, it has not yet been established as to which contribution is dominant.

Schmidt and Brown revealed that AGPAT3 regulated the structure and functions of the Golgi complex [74]. The overexpression of AGPAT3 significantly inhibited the formation of Golgi membrane tubules and also reduced anterograde and retrograde protein trafficking through the Golgi apparatus. In contrast, the reduced expression of AGPAT3 by siRNA accelerated protein trafficking. Golgi morphology was also dependent on AGPAT3 because its knockdown caused Golgi fragmentation. These findings clearly demonstrated a direct role for a specific acyltransferase in the regulation of membrane trafficking and organelle structures.

### 4.4. AGPAT4 (LPAATδ) and AGPAT5 (LPAATε)

AGPAT4 and AGPAT5 were also reported to be members of the AGPAT family of enzymes [70,71,72]. Mouse AGPAT4 mRNA levels were found to be very high in the brain, moderate in the lungs, intestines, kidneys, epidermis, and spleen, and low in the liver, heart, and adipose tissue. In contrast, mouse AGPAT5 mRNA levels were high in the brain, adipose tissue, and epidermis, moderate in the heart, kidneys, liver, and lungs, and low in the spleen. The prediction of topology by several programs suggested that AGPAT4 and AGPAT5 were located in mitochondria. Prasad* et al.* reported that the AGPAT5-GFP fusion protein was localized in the mitochondria of both CHO and human epithelial cervical cancer cells [72].

Mouse AGPAT4 possessed LPAAT activity with a significant preference for PUFA-CoA, especially DHA-CoA [77]. The high level of expression of AGPAT4 in the brain may correspond to DHA being abundant in brain phospholipids. The siRNA-mediated knockdown of AGPAT4 in Neuro 2A cells caused a decrease in 18:0–22:6 PC. AGPAT4 may play a significant role in the maintenance of DHA in neural membranes.

Although AGPAT5 exhibited LPAAT activity, this enzyme also displayed similar acyltransferase activity to other lysophospholipids including LPC, LPE, LPS, and LPI [72]. The LPEAT activity of AGPAT5 preferred C18:1 fatty acyl-CoA over C20:4-CoA. AGPAT5 has been suggested to be involved in both the *de novo* synthesis and remodeling of phospholipids; however, it has not yet been established which contribution is dominant. Prasad* et al.* proposed that the LPAAT activity of AGPAT5 may be important because of the localization of AGPAT5 in mitochondria; its co-localization with GPAT1 and GPAT2 in mitochondria supports mitochondria being capable of synthesizing PA, a precursor of their own glycerophospholipids.

## 5. Acylglycerophosphate Acyltransferases (AGPATs) Involved in the Remodeling of Phospholipids

Detailed substrate specificities using recombinant enzymes suggested that some AGPAT family enzymes possessed acyltransferase activity toward various lysophospholipids such as LPC. These findings suggested that some AGPAT enzymes were involved in the remodeling of phospholipids (Figure 1) [4,5,6,7,8].

### 5.1. LPCAT1/AGPAT9 

The acyltransferase for LPC (LPCAT) was identified to be a member of the AGPAT family [78,79,80,81]. Nakanishi* et al.* cloned mouse LPCAT1 and its human homolog based on sequence similarities to AGPATs [78]. Although this enzyme was named LPCAT1, another name ACYLTRANSFERASE-LIKE 2 (AYTL2) was also proposed [80]. LPCAT1 contains the acyltransferase motifs conserved in members of the AGPAT family. LPCAT1 was predicted to have three putative transmembrane domains, a putative EF hand domain, and C-terminal ER retention signal. LPCAT1 was shown to be mainly located in ER membranes [78]. It was expressed at its highest levels in the lungs, followed by the spleen and placenta, while other tissues had low levels, and was expressed at higher levels in isolated rat alveolar type II cells than in the whole lung [78,79,80,81].

LPCAT1 exhibited acyltransferase activity for LPC. LPCAT1 preferred saturated fatty acyl-CoAs as acyl donors and 1-myristoyl or 1-palmitoyl LPC as an acyl acceptor [78,81], suggesting that LPCAT1 was involved in di-saturated PC. The LPCAT activity of LPCAT1 was found to be Ca^2+^-independent; however, this enzyme possessed EF-hand-like motifs [78]. LPCAT1 also exhibited lower acyltransferase activity for other lysophospholipids such as LPA, LPE, and LPG [80]. LPCAT1 was inhibited by calcium and magnesium, which suggested that EF-hand-like motifs were involved in its inhibition.

Based on its high expression in alveolar type II cells and preference for the saturated species of acyl-CoA and LPC, this enzyme was suggested to be involved in the biosynthesis of di-saturated PC in pulmonary surfactant [78,79]. Chen* et al.* showed a correlation between the development of the prenatal lung and the expression of LPCAT1 [79]. Dexamethasone and KGF (FGF7), which are known to stimulate the synthesis of surfactant, significantly increased the expression of LPCAT1 in lung explants, whereas the inhibition of FGF signaling prevented this increase in LPCAT alveolar type II cells. These findings indicated that LPCAT1 played a critical role in the regulation of surfactant PC biosynthesis.

Studies on genetically manipulated mice indicated that newborn mice bearing a hypomorphic allele of *LPCAT1* showed varying levels of perinatal mortality from respiratory failure; *LPCAT1* mRNA levels were reduced and directly correlated with LPCAT1 activity, saturated PC content, and survival [82]. Surfactant isolated from dead mice failed to reduce minimum surface tension to wild-type levels. Collectively, these findings demonstrated that full LPCAT1 activity was required to achieve the levels of saturated PC essential for the transition to air breathing. Harayama* et al.* also generated Lpcat1-deficient mice [83]. Reduced dipalmitoyl PC in lung were confirmed in Lpcat1^−/−^ mice. They suggested that dipalmitoyl PC are important to prevent the early onset of acute lung injury.

The role of LPCAT1/AGPAT9 in the synthesis of platelet-activating factor (PAF) was proposed [13]. Harayama* et al.* reported that LPCAT1 recognized both lysoPAF and acetyl-CoA and catalyzed lysoPAF acetyltransferase reaction. They also proposed a novel noninflammatory biosynthetic pathway of PAF by LPCAT1.

Friedman* et al.* found that mouse mutants exhibiting rapid photoreceptor dysfunction, followed by the degeneration of both rods and cones, possessed nucleotide insertions or deletions in exons in the LPCAT1 gene that resulted in a frame-shift, leading to the premature truncation of the LPCAT1 protein [84]. An analysis of retinal lipids from mutant mice showed markedly lower dipalmitoyl PC levels than those in control mice. These results may suggest the essential role of LPCAT1 and the major product dipalmitoyl PC in retinal photoreceptor homeostasis.

The relationship between the *de novo* synthesis and remodeling of PC has been reported previously [85]. The ectopic overexpression of LPCAT1 decreased *de novo* PC synthesis by increasing the degradation of cholinephosphotransferase 1 (CPT1), which catalyzes the terminal step in *de novo* PC synthesis, without significantly altering cellular PC levels. The degradation of CPT1 is involved in its multi-ubiquitination and processing via the lysosomal pathway. These findings indicated that homeostatic control of the cellular content of PC was regulated by crosstalk between phospholipid remodeling and *de novo* pathways. In contrast, the degradation of LPCAT1 occurred with the treatment of murine lung epithelial cells with lipopolysaccharide in proteasomes following polyubiquitination of the enzyme [86]. The degradation of LPCAT1 may represent a unique strategy to optimize dipalmitoyl PC levels in sepsis.

Moessinger* et al.* reported that LPCAT1 was also localized in lipid droplets, which consist of a core of neutral lipids surrounded by a monolayer of phospholipids, mainly PC [87]. The activity of the remodeling pathway (LPCAT activity), but not the* de novo* synthesis of PC (CPT activity), was detected in lipid droplets and was correlated to the expression levels of LPCAT1, suggesting that LPCAT1 was also involved in PC synthesis in lipid droplets.

An additional function of LPCAT1 has been reported. Zou* et al.* found that histone H4 was subject to palmitoylation catalyzed by LPCAT1 in a calcium-regulated manner [88]. LPCAT1 in the cytoplasm was translocated to the nucleus in lung epithelia in response to exogenous Ca^2+^. Nuclear LPCAT1 colocalized with and bound to histone H4, at which it catalyzed histone H4 palmitoylation. These findings suggested that histone palmitoylation by LPCAT1 was a novel post-translational modification and this epigenetic modification regulated global gene transcriptional activity.

Another proposal was a diagnostic marker for the progression of cancer. The expression of LPCAT1 in tissue microarray slides containing samples from patients with prostatic disorders was the highest in metastatic prostate cancer, and was significantly higher than that in primary prostate cancer, high grade prostatic intraepithelial neoplasia, and the benign prostate [89]. The expression of LPCAT1 was also correlated to the tumor grade and stage of prostate cancer. Mansilla *et al.* reported that the expression of LPCAT1 was higher in colorectal adenocarcinomas than in normal mucosa [90]. The LPCAT1-overexpressing colon cancer cell line, SW480 significantly increased its growth rate by 17%.

### 5.2. LPCAT2/AGPAT11

Mouse LPCAT2/AGPAT11 was also identified based on sequence homology with the previously reported LPCAT1 [91]. Mouse LPCAT2 showed 48.2% amino acid sequence homology to mouse LPCAT1 and is also a member of the AGPAT family (AGPAT11). Although this enzyme was named LPCAT2, several other names, including ACYLTRANSFERASE-LIKE 1 (AYTL1), LysoPAFAT, and AGPAT11, have also been proposed [80,92]. Based on its amino acid sequence, LPCAT2 was predicted by the presence of three putative transmembrane domains, a putative EF-hand-like motif, and C-terminal ER retention signal. LPCAT2 is mainly located in ER membranes. Three splice variants of mouse LPCAT2 have been identified [80], with two variants (AYTL1_v2 and AYTL1_v3) lacking 40 amino acid residues including the conserved acyltransferase motif III (PEGT), which may be essential for catalytic activity and participate in binding to the acyl acceptor [9,10,11,12]. The mRNA of LPCAT2 was expressed at its highest levels in resident macrophages and activated neutrophils, followed by the skin, colon, spleen, and brain [91]. The expression of this enzyme in macrophages was augmented by Toll-like receptor agonists, while its induction was blocked by dexamethasone.

The substrate selectivity of LPCAT2 was examined, and the findings obtained demonstrated that this enzyme exhibited not only arachidonoyl-CoA acyltransferase, but also acetyl-CoA acetyltransferase activities toward 1-acyl or 1-alkyl LPC. Acetyltransferase (LysoPAFAT) activity was shown to be involved in the biosynthesis of PAF [91]. Under resting conditions, LPCAT2/LysoPAFAT preferred arachidonoyl-CoA to form the precursor of PAF (1-*O*-alkyl-2-arachidonoyl PC), while the activated enzyme used acetyl-CoA more efficiently upon acute inflammatory stimulation with LPS and produced PAF. LPCAT2/LysoPAFAT was dually regulated in both transcriptional and post-translational processes [93,94]. The mRNA of LPCAT2 was induced by inflammatory stimuli, whereas the LPS treatment enhanced LysoPAFAT activity by phosphorylating the enzyme. LPS induced the phosphorylation of Ser34 in LPCAT2 and the involvement of MAPK cascades was confirmed [93]. The phosphorylation of Ser34 also occurred by the stimulation of GPCRs; Ser34 phosphorylation induced a rapid biosynthesis of PAF and was mediated by PKCα-signaling [94]. These findings provided an important insight into the regulation of PAF synthesis.

Since PAF is a potent pro-inflammatory mediator, the chemicals that inhibit PAF synthesis are of therapeutic interest. Several inhibitors of LPCAT2 were recently developed [95,96]. Yamazaki* et al.* isolated an inhibitor, dihydrofumigatin (2-methoxy-1,3,4-trihydroxy-5-methylbenzene), in a metabolite from *Penicillium* *sp. F33* [95]. They also chemically synthesized its oxidized form fumigatin (3-hydroxy-2-methoxy-5-methyl-1,4-benzoquinone) and derivatives. The compounds showed anti-inflammatory activity* in vivo* using the carrageenan-induced mouse paw edema test. Tarui* et al.* also isolated LPCAT2-specific inhibitors, N-phenylmaleimide derivatives, selected from a 174,000-compound library [96]. The compounds competed with acetyl-CoA for the inhibition of LPCAT2 and suppressed PAF biosynthesis in mouse peritoneal macrophages. The compounds had low inhibitory effects on LPCAT1 activity, indicating that adverse effects on respiratory functions may be avoided. These compounds will contribute to the development of novel drugs for PAF-related diseases and facilitate the analysis of LPCAT2 functions in phospholipid metabolism* in vivo*.

Similar to LPCAT1, LPCAT2 was also shown to localize in lipid droplets, suggesting that LPCAT2 was also involved in PC synthesis in lipid droplets and in lipid storage [87]. Bouchoux* et al.* also identified LPCAT2 as a lipid droplet-associated protein in differentiated Caco-2/TC7 enterocytes [97]. LPCAT2 in enterocytes may provide PC, which is necessary for chylomicron assembly, from LPC present in the intestinal lumen.

Agarwal and Garg reported the AGPAT activity of AGPAT11/LPCAT2 and increased expression of AGPAT11 in breast cancer and cervical cancer tissues [92]. The expression of AGPAT11/ LPCAT2 reflected the tumor grade. They also found the increased expression of AGPAT11/LPCAT2 in colorectal carcinoma tissues. They proposed that an elevation in the level of PA produced by AGPAT11 may be involved in cancer progression because PA can activate and amplify Ras signaling, resulting in MAP kinase and PI3K/AKT for survival pathways for the neoplastic anchorage-independent survival of tumors [98,99,100].

### 5.3. AGPAT7/LPEAT2

AGPAT7 was first identified by searching an EST database for sequences similar to LPAAT-zeta [34,101]. The enzyme protein composed by the deduced 524 amino acids was shown to have a calculated molecular mass of 57.2 kDa. The expression of AGPAT7 has been detected in the uterus, thymus, pancreas, skeletal muscle, bladder, stomach, lungs, and testis. AGPAT7 mainly localized in ER. Soupene* et al.* reported this enzyme as ACYLTRANSFERASE-LIKE 3 (AYTL3) [80]. This enzyme is closely related to two LPCATs (LPCAT1/AGPAT9/ACYL2 and LPCAT2/LysoPAFAT/AGPAT11/ACYL1). Recombinant mouse Aytl3 in *E. coli* membranes exhibited acyltransferase activity for LPC, but not for the other lysophospholipids tested, in the presence of various acyl-CoA donors. However, Cao* et al.* described AGPAT7 as an acyltransferase that exhibited prominent activity toward ethanolamine-containing lysophospholipids, including 1-acyl LPE and 1-*O*-alkenyl LPE, and was consequently renamed LPEAT2 [102]. AGPAT7/LPEAT2 recognizes a broad range of medium- and long-chain fatty acyl-CoA. The siRNA-mediated knockdown of AGPAT7/LPEAT2 in HEK293T cells significantly decreased LPEAT and 1-alkenyl LPEAT activities, but did not affect other lysophospholipid acylating activities. LPEAT2 was predominantly expressed in the brain, coinciding with the enrichment of PE in this tissue. These findings suggested that LPEAT2 was an important enzyme in the biosynthesis and remodeling of ethanolamine-containing glycerophospholipids, especially in the brain.

### 5.4. AGPAT8/ALCAT1/2-Acyl LPIAT

AGPAT8 was first identified as mouse acyl-CoA:lysocardiolipin acyltransferase 1 (ALCAT1) [103]. Recombinant mouse ALCAT1 exhibited acyltransferase activities toward both monolysocardiolipin (MLCL) and dilysocardiolipin (DLCL), but no significant activities for either G3P, LPC, LPE, or LPS. Cao* et al.* suggested that ALCAT1 was involved in the remodeling of cardiolipin (CL) to maintain heart function since the ALCAT1 gene was highly expressed in the heart, in which CL is present. However, mouse ALCAT1 was shown to localize in the ER, in which the location of the enzyme did not correspond to CL being a mitochondrion-specific phospholipid.

Li* et al.* reported that the remodeling of CL by ALCAT1/AGPAT8 was involved in mitochondrial dysfunction and susceptibility to diet-induced obesity [104]. The ALCAT1 protein was predominantly localized in mitochondria-associated membranes, a subfraction of the ER. The overexpression of ALCAT1 in C2C12 cells reduced total CL levels by selectively decreasing the content of C16-C18 fatty acids in CL, while DHA (C22:6 n-3)-containing CL species increased by more than 300%. Consistent with the increased DHA content in CL, the overexpression of ALCAT1 caused mitochondrial dysfunction by significantly increasing the mitochondrial membrane potential, oxygen consumption rate, and proton leakage. In addition, ALCAT1 was up-regulated by oxidative stress and diet-induced obesity, which indicated that ALCAT1 synthesized CL species with PUFA that were highly sensitive to oxidative damage, leading to mitochondrial dysfunction, reactive oxygen species production, and insulin resistance. Consequently, the ALCAT1 deficiency prevented the onset of diet-induced obesity and significantly improved mitochondrial complex I activity, lipid oxidation, and insulin signaling in ALCAT1^−/−^ mice. In support of the regulatory role of ALCAT1/AGPAT8 in CL remodeling in response to oxidative stress, the expression of ALCAT1 in the liver and heart was significantly down-regulated in mice with hypothyroidism and up-regulated in mice treated with thyroid hormone, which is known to stimulate mitochondrial activity, oxidative stress, and CL remodeling. These findings suggested the key role of ALCAT1 in mitochondrial dysfunction associated with hypertrophic cardiomyopathy [105,106].

The multiple functions of AGPAT8/ALCAT1 were proposed based on detailed substrate specificities [103,104,105,106,107]. Zhao* et al.* found that human AGPAT8/ALCAT1 also possessed acyltransferase activities toward LPI and LPG [108]. The siRNA-mediated knockdown of ALCAT1 in Hela cells resulted in significant reductions in LPIAT and LPGAT activities, but not in ALCAT activity, suggesting that the physiological substrates of ALCAT1 were LPI or LPG, but not MLCL. Cao* et al.* also reported that AGPAT8/ALCAT1 catalyzed the acylation of bis(monoacylglycero)phosphate, a structural isomer of PG that is located in endosomes [105]. These findings suggested that AGPAT8/ALCAT1 played a role in the remodeling of other polyglycerophospholipids, and was also supported by ALCAT1 being localized in the ER, but not in mitochondria in which CL is located. In contrast, Agarwal* et al.* reported that AGPAT8 had moderate acyltransferase activity for LPA with oleoyl-CoA, but lacked acyl-CoA:MLCL acyltransferase activity [107]. The overexpression of AGPAT8/ALCAT1 in whole cells upon incubation with [^14^C] linoleic acid augmented the incorporation of radioactivity in PE, PC, and PA, but not in CL.

An analysis of the yeast and* C. elegans* homologs of AGPAT8/ALCAT1 suggested that the physiological substrates of ALCAT1 were 2-acyl LPI. LeGuedard* et al.* reported that the disruption of a yeast homolog of AGPAT8/ALCAT1 caused the almost complete disappearance of stearic acid (18:0) at the *sn*-1 position of PI [109]. The mutant was devoid of acyltransferase activity for *2*-acyl LPI, and this activity was recovered by the expression of the enzyme named Psi1p (phosphatidylinositol stearoyl incorporating 1 protein) in the mutant. Imae* et al.* also reported that orthologs of AGPAT8/ALCAT1 in *C. elegans* were involved in the *sn*-1 fatty acid remodeling of PI [110]. The 18:0 content of PI was reduced in *C. elegans* mutants, and both recombinant *C. elegans* and mouse AGPAT8 exhibited stearoyl-CoA acyltransferase activity for 2-acyl LPI. Imae* et al.* generated AGPAT8-deficient mice and demonstrated that AGPAT8 determined the fatty acid composition of PI in mammals [111]. These findings indicated that AGPAT8/ALCAT1 were involved in the *sn-*1 stearic acid remodeling of PI and, furthermore, that the physiological substrates of ALCAT1/AGPAT8 may be 2-acyl LPI.

Mouse AGPAT8/ALCAT1 was abundantly expressed in hematopoietic stem cells, which undergo asymmetric divisions to renew themselves and produce various progeny cells of distinct blood lineages [112]. The knockdown of zebrafish AGPAT8/ALCAT1 resulted in a reduction in the number of blood cells [113]. In addition, *C. elegans* AGPAT8/ALCAT1 was also shown to be involved in the asymmetric division of stem cell-like epithelial cells [110,111]. The remodeling of CL or *sn*-1 stearic acid remodeling of PI may be involved in an evolutionarily conserved role of the *sn*-1 fatty acid of PI in the asymmetric division of stem cells. 

### 5.5. Tafazzins and the Fatty Acid Remodeling of Cardiolipin

Another remodeling enzyme of CL was identified by the analysis of an inherited disorder. Barth Syndrome is a severe inherited disorder that is often fatal in childhood, and is characterized by cardiac and skeletal myopathy, a short stature, and neutropenia [114]. Mutations in the gene, termed G4.5, were identified in the distal portion of Xq28, and the G4.5 gene was expressed at high levels in cardiac and skeletal muscle [115]. Mutations introduced stop codons in the open reading frame, which interrupted translation of the putative protein “tafazzin (TAZ)”. An abnormal fatty acid composition was detected in CL from patients with Barth syndrome [116]. The deficiency of tetralinoleoyl CL, a major species in heart and skeletal muscle, was observed in those from patients. Collectively, these findings suggested that the G4.5 gene was the genetic locus responsible for Barth syndrome and TAZ was a putative acyltransferase involved in the incorporation of C18:2*n*-6 into CL. Furthermore, the normal fatty acid composition of CL may be important for the biological functions of CL, in particular, for mitochondrial functions.

Schlame and co-workers examined the enzymatic activities of *Drosophila melanogaster* TAZ [117,118,119]. The recombinant TAZ catalyzed fatty acid transfer between CL and PC. CoA and acyl-CoA were not involved in transacylation reactions. Transacylation activities were approximately 10-fold higher for linoleoyl groups than for oleoyl groups and were negligible for arachidonoyl groups. Transacylation activity was shown to be decreased in lymphoblasts from patients with Barth syndrome [117,118]. These findings indicated that human and *Drosophila* TAZs were a CoA-independent, acyl-specific phospholipid transacylase with substrate preference for CL and PC. 

Soustek* et al.* generated transgenic mice that inducibly expressed shRNA for TAZ knockdown [120]. TAZ deficiencies led to the absence of tetralineoyl-CL, accumulation of MLCL, and alternation in mitochondrial morphology in cardiac muscle. Physiological measurements demonstrated that the isometric contractile strength of the soleus muscle and cardiac left ventricular ejection fraction were lower in TAZ-depleted mice. These findings suggested that transgenic mice exhibited the same symptoms as Barth Syndrome and confirmed that TAZ was the enzyme responsible for the genetic disease.

### 5.6. Lysophosphatidylglycerol Acyltransferase 1 (LPGAT1)

Yang* et al.* identified and characterized a human gene encoding an acyl-CoA:LPG acyltransferase (LPGAT1) [121]. Recombinant human LPGAT1 was localized to the ER and exhibited acyltransferase activity for LPG, but not for G3P, LPC, LPE, LPI, or LPS. LPGAT1 demonstrated a clear preference for long-chain saturated 16:0- or 18:0-CoAs and oleoyl-CoA as acyl donors, which was consistent with the composition of endogenous PGs in tissues.

Hiramine* et al.* found that mouse LPGAT1 exhibited MGAT activity and was involved in the synthesis of TAG [122]. Hepatic LPGAT1 expression in diabetic *db*/*db* mice was higher than that in control *db*/*m* mice, which was consistent with the increase in hepatic MGAT activity observed in *db/db* mice. The knockdown of LPGAT1 in the livers of *db*/*db* mice by an adenovirus of shRNA for LPGAT1 significantly reduced hepatic MGAT activity and serum TAG/cholesterol levels. These findings indicated that LPGAT1, another MGAT enzyme, played a significant role in the synthesis and secretion of TAG in *db/db* mice.

## 6. Conclusions

In this review, we described recent advances in research on GPAT/AGPAT family enzymes. Large numbers of GPAT/AGPAT family enzymes were cloned based on the sequences of *E. coli* GPAT/AGPAT. Detailed enzymology revealed that GPAT/AGPAT enzymes, in particular, GPAT1, GPAT4, and AGPAT2, are involved in the storage of TAG and obesity-related diseases. The identification of causative genes and analysis of gene-manipulated animals also support these findings. We propose that these enzymes should be the candidates of therapeutic targets for obesity-related diseases. Inhibitors of GPAT3, possibly mediating the prevention of adipocyte hypertrophy, might be used in the treatment of diabetes. In addition to the roles in synthesis and storage of TAG, the sequential reactions of GPAT/AGPAT are involved in the synthesis of intracellular messenger PA, and the inhibitors of AGPAT2 are suggested as candidates for anti-cancer drugs.

In addition, detailed enzymology revealed that some isoforms of AGPAT including AGPAT9/LPCAT1, AGPAT11/LPCAT2, and AGPAT8/ALCAT1 exhibit acyltransferase activity towards other lysophospholipids, suggesting that these isoforms are involved in the fatty acid remodeling of phospholipids. Since many other enzymes and enzymatic reactions including MBOATs, phospholipases, and transacylation reactions, are involved in the remodeling, the relationship and different roles of AGPATs and these enzymes in this remodeling must be explored. Further studies are thus necessary to better understand the physiological roles of fatty acid remodeling of phospholipids.
